# First Detection of *Aedes* (*Stegomyia*) *albopictus* (Diptera: Culicidae) in Algiers, the Capital City of Algeria

**Published:** 2019-12-31

**Authors:** Kamel Eddine Benallal, Rafik Garni, Lazhari Bouiba, Zoubir Harrat

**Affiliations:** Laboratoire d’Eco-épidémiologie Parasitaire et Génétique des Populations, Institut Pasteur d’Algérie, Route Petit Staouéli, Dely Ibrahim, Alger, Algérie

**Keywords:** *Aedes albopictus*, Mosquitoes, Arbovirus, Algiers, Algeria

## Abstract

**Background:**

Based on the reporting of the presence of stripped mosquitoes by a citizen in the Algiers residential neighborhood of Bir-Khadem, where residents experienced huge daytime mosquito nuisance an entomological investigation was carried out in July 2016.

**Methods:**

Ovitraps and BG sentinel traps baited with Lure were used during three consecutive days to collect adult mosquitoes. Eighteen residential houses of the Bir-Khadem neighborhood were also inspected to search larvae breeding sites such as water fountains, baskets and flowerpots.

**Results:**

A total of 57 *Aedes albopictus* specimens were collected in five villas, consisting of 21 eggs, 20 larvae and 16 adults.

**Conclusion:**

This is the first record of this invasive species in Algiers.

## Introduction

*Aedes albopictus* is currently considered the most invasive mosquito species (1) as its rapid adaptation to a large variety of environments in both tropical and temperate areas has allowed its spread throughout the world (2). Indeed, *Ae. albopictus* larvae can breed in different container forms (3). As early as 1968, *Ae. albopictus* was reported as the most common and widespread mosquito in Southeast Asia (4). In 1979, this mosquito was reported in Albania (5) then in several countries in Europe, around the Mediterranean basin and in Morocco (6, 7). Doubtlessly, the worldwide dispersion of *Ae. albopictus* is mainly caused either by trade in used tires or lucky bamboo between the continents and by travelers and cars within land (8, 9).

Beside its huge nuisance, *Ae*. *albopictus* capacity to spread could engender a serious threat for the public health since it is competent to transmit several viruses (10). This mosquito has been responsible for the large out-break of Chikungunya in La Réunion Island and focal transmission of Dengue and Chikungunya in Europe (11–13). Since then because of its ability to adapt to different environments, this mosquito species is under scrutiny by many health services. It has also been shown to be competent for Zika virus (10, 14) but its role in the current Zika epidemics needs further investigation. *Aedes albopictus* is also known as a vector for West Nile virus (15, 16), *Dirofilaria immitis* and *D. repens* (17, 18). In Algeria, it was reported on three occurrences (19–21), but never in Algiers, the capital city of Algeria.

In July 2016, a citizen contacted our laboratory to complain from strange mosquitoes that caused painful bites and huge daytime nuisance. The herein entomological survey led to the identification of *Ae. albopictus* in the district of Bir-Khadem and assessed its spread in the district.

## Materials and Methods

Bir-Khadem is a locality of Algiers (altitude 84m, 36.716460° N, 3.042561° E), population number 77. 749 persons located about 6km from the commercial port and 16km off the international airport. In Algiers, Mediterranean climate occurs with a mean 686mm rainfall per year and a mean temperature of 23 °C.

A citizen living in a residential neighborhood brought to our laboratory some mosquito specimens collected in his house. These mosquitoes bite during daytime and made a huge nuisance, which was suggestive of *Ae. albopictus*. Following this, sampling procedures for detecting invasive mosquitoes were implemented in that neighborhood.

Two Biogents-Sentinel™ trap (BGS) (Biogents AG, Regensburg, Germany) baited with BG-Lure (BGL) a mosquito attractive were run outside in the gardens (first at the house of the complainer and the second at the house of the Asian persons) for three consecutive days to collect adult mosquitoes. A total of 18 residential houses of the Bir-Khadem neighborhood were also inspected in order to search possible larval breeding sites such as water fountains, baskets and flowerpots plates. Ovit-raps consisting of black plastic container filled with 400ml of water and leaves provided with a 20× 20× 5cm of polyester piece as a substrate for *Aedes* spp. mosquito oviposition device were placed under trees and in shaded areas (11). The collected adult and larvae specimens were morphologically identified (22).

A survey with local citizens (18 households) about the first appearance of this mosquito in the area was carried out mainly was around: i) the period of biting and activity of this species (daytime or night), ii) what is new in the neighborhood (the installation of new people, a new commercial activity especially that of used tires or the sale of exotic plants), iii) the notion of travel in countries endemic to *Ae. albopictus*. All these questions were asked in order to find an explanation how this mosquito has came to this area since it was reported established only in Oran which is distanced about 500km from Algiers (21).

## Results

**Fig. 1.**
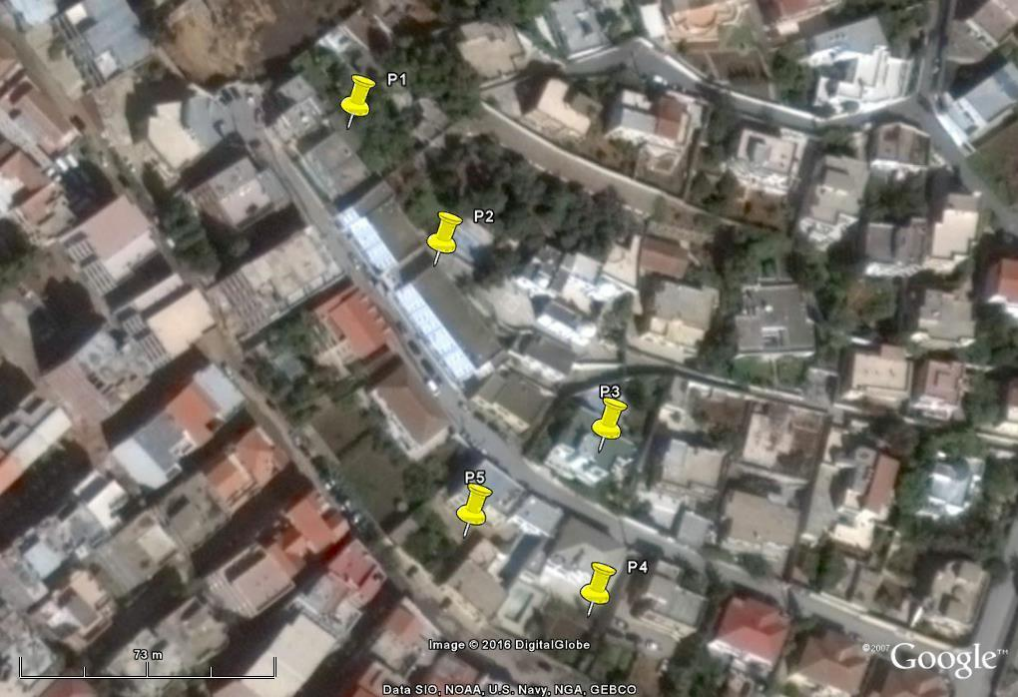
Location of *Aedes albopictus* captures at different development stages

The eight mosquitoes (two males and six females) collected by the citizen belonged to Aedes genus showed black and white stripes showed black and white stripes on their legs and a medio-dorsal white stripe on the thorax characteristic of *Ae. albopictus*. Out of the 18 residential houses prospected 4 were positives with *Ae. albopictus* in association with *Culex pipiens* and *Culiseta longiareolata* around the Asian foreign villas (Table 1, Fig. 1). A total of fifty-seven adults of *Ae. albopictus* (male and female) were identified either collected at adult stages or emerged from larvae and nymphs.

**Table 1.** Location and stage of *Aedes albopictus* collected during the survey

**House**	**Specimen numbers**		

	**Adultes**	**Larvae**	**Eggs**
**P1**	13	15	7
**P2**	11	/	/
**P3**	/	/	6
**P4**	/	5	/
**Total**	24	20	13

P1: villa where first specimens were collected, P2: foreign’s villa, P3: second positive villa, P4: third positive villa

The survey carried out with local citizens revealed that this species bites in daytimes and had appeared one month before in June and coincided with the installation of Asian persons who work for a building company in Algiers. They had not reported new commercial activities (tires or plants) since it was a residential quarter. Concerning travelling notion no one had been in an endemic area of *Ae. albopictus*.

## Discussion

An entomological survey was implemented in a residential neighborhood of Bir-Khadem in order to identify the mosquito species implicated in the source of nuisance. As suspected, several specimens of *Ae. albopictus* were found. Residential houses with garden, swimming pool, vegetation, trees and animals provide suitable environmental conditions for this species to reproduce. In addition, the stability of good climate conditions during June till August 2016 (mean temperature 25 °C) has allowed *Ae. albopictus* to reproduce and colonize easily this area. The occurrence of this species in this locality with mean annual precipitation of 686 mm is of great interest since over most of its range is associated with very heavy rainfall (23).

BG-Sentinel traps and ovitraps allow the collection of few specimens only. This suggests that *Ae. albopictus* population is still at low level despite favorable environmental conditions or at the beginning of its introduction. Besides, the questionnaire survey carried out with the citizens revealed that this mosquito had appeared since June 2016. One might wonder whether the source of *Ae*. *albopictus* could be some eggs carried in the plants that they brought from their country and cultivated in their garden for their own consummation since no commercial activity nor tire manufactory are registered in this area.

*Aedes albopictus* is well established all around the Mediterranean basin and risk-mapping modeling showed that Northern Algeria is suitable for its installation (24). Since the report of dengue and Chikungunya in Europe (25, 26) and several cases of West Nile in Algeria (27, 28), the Algerian Government has established an arbovirus surveillance system according to the International Health Regulation. Unfortunately, the surveillance system setup has not allowed the early detection of *Ae*. *albopictus* introduction into Algeria but thanks to the vigilance of inhabitants who reported these mosquitoes which proves that implication of citizens remains the most important way to report and monitor the establishment of invasive mosquitoes (11). Even if insecticide (Allethrin) spraying was performed by Algiers Board of Health to eliminate adults before possible establishment, a continuous surveillance with ovitraps and BG-sentinel traps is highly recommended to monitor its extension area since *Ae*. *albopictus* is known to skip oviposition and to release its eggs in several breeding sites (29).

A good communication campaign in radio and television should be also planned as it is a new mosquito species with new behavior in order to explain to citizens how avoiding the development of this species in their garden and how to limit its propagation by simple gestures like covering the water barrels, emptying the flowerpot plates, removing unnecessary utensils from the gardens and the surrounding areas that may contain water and help the pullulation of these mosquitoes. Such measures need to be planned before for limiting *Ae. albopictus* propagation to other areas and to avoid the massive using of insecticides for vector control which could favor the appearance of resistance with time.

## Conclusion

The fourth record of *A. albopictus* in Algeria suggests that the surveillance system efficiency must be improved by sensitizing and implicating population awareness for this mosquito species in time. Furthermore, involvement of *Ae. albopictus* in the transmission of Dengue, Chikungunya, and Zika viruses from viremic persons highlights the importance of monitoring this invasive species to assess the health risk. Further studies are recommended to determine the origin of the specimens collected in Algiers, and to investigate the ecology and sensitivity to insecticides of this species in case it establishes in Algiers for planning adapted and efficient control measures.

## References

[1] Enserink M (2008) A mosquito goes global. Science. 320: 864–866. 18487167 10.1126/science.320.5878.864

[2] Gould EA Higgs S (2009) Impact of climate change and other factors on emerging arbovirus diseases. Trans R Soc Trop Med Hyg. 103: 109–121. 18799177 10.1016/j.trstmh.2008.07.025 PMC2915563

[3] Hawley WA Reiter P Copeland RS Pumpuni CB Craig GB JR (1987) *Aedes albopictus* in North America: probable introduction in used tires from northern Asia. Science. 236: 1114–1116. 3576225 10.1126/science.3576225

[4] Huang YM (1968) Neotype designation for *Aedes* (Stegomyia) *albopictus* (Skuse) (Diptera: Culicidae). Proc Ent Soc Wash. 70: 297–302.

[5] Adhami J Reiter P (1998) Introduction and establishment of *Aedes* (*Stegomyia*) *albopictus* Skuse (Diptera: Culicidae) in Albania. J Am Mosq Control Assoc. 14: 340–343. 9813831

[6] Technical report: Development of *Aedes albopictus* risk maps (2009) ECDC, Stockholm Available at: http://ecdc.europa.eu/en/healthtopics/vectors/vector-maps/Pages/VBORNET_maps.aspx.

[7] Bennouna A Balenghien T Rhaffouli H Schaffner F Garros C Gardès L Lhor Y Hammoumi S Chlyeh G Fassi-Fihri O (2016) First record of *Stegomyia albopicta* (= *Aedes albopictus*) in Morocco: a major threat to public health in North Africa? Med Vet Entomol. 31(1): 102–106. 27775162 10.1111/mve.12194

[8] Pluskota B Storch V Braunbeck T Beck M Becker N (2008) First record of *Stegomyia albopicta* (Skuse) (Diptera: Culicidae) in Germany. Euro Mosq Bull. 26: 1–5.

[9] Müller GC Tsabari O Traore MM Traore SF Doumbia S Kravchenko VD Junnila A Beier JC (2016) First record of *Aedes albopictus* in inland Africa along the River Niger in Bamako and Mopti, Mali. Acta Trop. 162: 245–247. 27450393 10.1016/j.actatropica.2016.07.008 PMC4989851

[10] Glida G Caron M Mombo IM Nkoghe D Ondo SM Davy J Fontenille D Paupy C Leroy EM (2014) Zika Virus in Gabon (Central Africa) –2007: A New Threat from Aedes albopictus? PLoS Negl Trop Dis. 8(2): e2681. 24516683 10.1371/journal.pntd.0002681 PMC3916288

[11] Barceló C Bengoa M Monerris M Molina R Delacour-Estrella S Lucientes J Miranda MA (2015) First record of *Aedes albopictus* (Skuse, 1894) (Diptera: Culicidae) from Ibiza (Balearic Islands; Spain). J Euro Mosq Control Assoc. 33: 1–4.

[12] Franke F Septfons A Leparc-Goffart I Giron S Guinard A Burdet S (2016) Surveillance du chikungunya, de la dengue et des infections à virus Zika en France métropolitaine 2017. Bull Epidémiol Hebd. 2017(12): 222–231

[13] Giovanni R (2018) Chikungunya is back in Italy: 2007–2017. J Travel Med. 25: 1–4. 10.1093/jtm/tay004 29669058

[14] Di Luca M Severini F Toma L Boccolini D Romi R Remoli ME Sabbatucci M Rizzo C Venturi G Rezza G Fortuna C (2016) Experimental studies of susceptibility of Italian *Aedes albopictus* to Zika virus. Euro Surveill. 21(18): 10.2807/1560-7917.ES.2016.21.18.30223. 10.2807/1560-7917.ES.2016.21.18.30223 27171034

[15] Medlock JM Hansford KM Schaffner F Versteirt V Hendrickx G Zeller H Bortel WV (2012) A Review of the Invasive Mosquitoes in Europe: Ecology, Public Health Risks, and Control Options Vector-Born Zoo Diseas. 12(6): 435–447. 10.1089/vbz.2011.0814 PMC3366101 22448724

[16] Fortuna C Remoli ME Severini F Di Luca M Toma L Fois F Bucci P Boccolini D Romi R Ciufolini MG (2015) Evaluation of vector competence for West Nile virusin Italian *Stegomyia albopicta* (=*Aedes albopictus*) mosquitoes. Med Vet Entomol. 29: 430–433. 26382099 10.1111/mve.12133

[17] Cancrini G Frangipane Di Regalbono AF Ricci I Tessarin C Gabrielli S Pietrobelli M (2003) *Aedes albopictus* is a natural vector of *Dirofilaria immitis* in Italy. Vet Parasitol. 118: 195–202. 14729167 10.1016/j.vetpar.2003.10.011

[18] Cancrini G Scaramozzino P Gabrielli S Di Paolo M Toma L Romi R (2007) *Aedes albopictus* and *Culex pipiens* implicated as natural vectors of *Dirofilaria repens* in central Italy. J Med Entomol. 44(6): 1064–1066. 18047207 10.1603/0022-2585(2007)44[1064:aaacpi]2.0.co;2

[19] Izri A Bitam I Charrel RN (2011) First entomological documentation of *Aedes* (Stegomyia) *albopictus* (Skuse, 1894) in Algeria. Clin Microbiol Infect. 17: 1116–8. 21435096 10.1111/j.1469-0691.2010.03443.x

[20] Lafri I Bitam I Beneldjouzi A Ben Mahdi MH (2014) An inventory of mosquetoes (Diptera: Culicidae) in Algeria. Bull Soc Zool Fr. 139(1–4): 255–261.

[21] Benallal KE Allal-Ikhlef A Benhamouda K Schaffner F Harra Z (2016) First report of *Aedes* (*Stegomyia*) *albopictus* (Diptera: Culicidae) in Oran, West of Algeria. Acta Trop. 164: 411–413. 27697483 10.1016/j.actatropica.2016.09.027

[22] Schaffner F Angel G Geoffroy B Hervy JP Rhaiem A Brunhes J (2001) The Mosquitoes of Europe /Les moustiques d'Europe. Logiciel d'identification et d'enseignement (CD-Rom), Montpellier, France, IRD Editions and EID Méditerranée.

[23] Qutubuddin M (1960) The mosquito fauna of Kohat-Hangu valley, West Pakistan. Mosquito News. 20(40): 355–361.

[24] Kraemer MUG Sinka ME Duda KA Mylne AQN Shearer FM Barker CM Moore CG Carvalho RG Coelho GE Van Bortel W Hendrickx G Schaffner F Elyazar IR Teng HJ Brady OJ Messina JP Pigott DM Scott TW Smith DL Wint GR Golding N Hay SI (2015) The global distribution of the arbovirus vectors *Aedes aegypti* and *Ae. Albopictus*. Elife. 4: e08347. 26126267 10.7554/eLife.08347 PMC4493616

[25] Rezza G Nicoletti L Angelini R Romi R Finarelli AC Panning M Cordioli P Fortuna C Boros S Magurano F Silvi G Angelini P Dottori M Ciufolini MG Majori GC Cassone A CHIKV study group (2007) Infection with CHIKV in Italy: an outbreak in a temperate region. Lancet. 370 (9602): 1840–6. 18061059 10.1016/S0140-6736(07)61779-6

[26] La Ruche G Dejour-Salamanca D Debruyne M Leparc-Goffart I Ledrans M Grandadam M Brichler S Souares Y Denoyel GA Poveda JD Gastellu-Etchegorry M (2010) Surveillance par les laboratoires des cas de dengue et de chikungunya importés en France métropolitaine 2008–2009. BEH. 31–32: 325–329.

[27] Santé publique France Institut de Veille Sanitaire (2012) Bulletin épidémiologique hebdomadaire. 10–16 10 2012 p. 369.

[28] Lafri I Hachid A Bitam I (2018) West Nile virus in Algeria: a comprehensive overview. New Microbes New Infect. 27: 9–13. 30519477 10.1016/j.nmni.2018.10.002 PMC6260397

[29] Davis TJ Kaufman PE Hogsette JA Kline DL (2015) The effects of larval habitat quality on *Aedes albopictus* skip oviposition. J Am Mosq Control Assoc. 31 (4): 321–328. 26675453 10.2987/moco-31-04-321-328.1

